# Benthic Carbon Mineralization and Nutrient Turnover in a Scottish Sea Loch: An Integrative In Situ Study

**DOI:** 10.1007/s10498-016-9300-8

**Published:** 2016-09-08

**Authors:** Ronnie N. Glud, Peter Berg, Henrik Stahl, Andrew Hume, Morten Larsen, Bradley D. Eyre, Perran L. M. Cook

**Affiliations:** 1 Nordic Centre for Earth Evolution, University of Southern Denmark, Odense, Denmark; 2 Scottish Marine Institute, Scottish Association for Marine Science, Oban, UK; 3 Arctic Research Centre, University of Aarhus, Aarhus, Denmark; 4 Department of Environmental Sciences, University of Virginia, Charlottesville, VA USA; 5 Zayed University, Dubai Academic City, Dubai, United Arab Emirates; 6 Centre for Coastal Biogeochemistry Research, Southern Cross University, Lismore, NSW Australia; 7 Water Studies Centre, School of Chemistry, Monash University, Clayton, VIC 3800 Australia

**Keywords:** Carbon cycle, Oxygen exchange, Nutrient regeneration, Denitrification, In situ measurements, Eddy covariance, Loch Etive

## Abstract

Based on in situ microprofiles, chamber incubations and eddy covariance measurements, we investigated the benthic carbon mineralization and nutrient regeneration in a ~65-m-deep sedimentation basin of Loch Etive, UK. The sediment hosted a considerable amount of infauna that was dominated by the brittle star *A. filiformis*. The numerous burrows were intensively irrigated enhancing the benthic in situ O_2_ uptake by ~50 %, and inducing highly variable redox conditions and O_2_ distribution in the surface sediment as also documented by complementary laboratory-based planar optode measurements. The average benthic O_2_ exchange as derived by chamber incubations and the eddy covariance approach were similar (14.9 ± 2.5 and 13.1 ± 9.0 mmol m^−2^ day^−1^) providing confidence in the two measuring approaches. Moreover, the non-invasive eddy approach revealed a flow-dependent benthic O_2_ flux that was partly ascribed to enhanced ventilation of infauna burrows during periods of elevated flow rates. The ratio in exchange rates of *Σ*CO_2_ and O_2_ was close to unity, confirming that the O_2_ uptake was a good proxy for the benthic carbon mineralization in this setting. The infauna activity resulted in highly dynamic redox conditions that presumably facilitated an efficient degradation of both terrestrial and marine-derived organic material. The complex O_2_ dynamics of the burrow environment also concurrently stimulated nitrification and coupled denitrification rates making the sediment an efficient sink for bioavailable nitrogen. Furthermore, bioturbation mediated a high efflux of dissolved phosphorus and silicate. The study documents a high spatial and temporal variation in benthic solute exchange with important implications for benthic turnover of organic carbon and nutrients. However, more long-term in situ investigations with like approaches are required to fully understand how environmental events and spatio-temporal variations interrelate to the overall biogeochemical functioning of coastal sediments.

**Electronic supplementary material:**

The online version of this article (doi:10.1007/s10498-016-9300-8) contains supplementary material, which is available to authorized users.

## Introduction

The coastal zone interfaces the terrestrial and oceanic biomes and is generally characterized by intense biogeochemical cycling. Riverine run-off and occasional upwelling supply nutrition for intense biological production and despite only covering ~7 % of the ocean bed, coastal sediments are globally important sites for organic carbon mineralization, nutrient regeneration and carbon preservation (Nixon et al. 1996; Gattuso et al. 1998; Wollast 1993; Smith et al. 2015). However, coastal sediments are also characterized by an extensive temporal and spatial variability that complicate the assessment on their biogeochemical function and importance. Daily, seasonal and interannual variations in hydrography and weather strongly influence deposition, particle dynamics and near-bed hydrodynamics that all directly or indirectly regulate the relative importance of the respective diagenetic pathways and the total benthic mineralization rate (Thamdrup et al. 1994; Cathalot et al. 2010; Liu et al. 2014). Additionally, particulate deposition and fauna activity can induce considerable microscale spatial variation in the intensity and the nature of the dominant microbial processes (Thouzeau et al. 2007; Dedieu et al. 2007; Glud et al. 2009a; Aller 2014). The intense metabolic activity also induces steep concentration gradients of redox species, and this together with the dynamic nature of coastal environments challenges procedures for quantifying and characterizing the early diagenesis. While laboratory-based investigations can provide valuable insight on diagenetic processes, measurements of benthic solute exchange using cores and microcosms can be compromised by disturbances during sediment recovery and challenges in re-establishing natural environmental conditions in the laboratory. However, while there for some time has been considerable awareness on the necessity of in situ investigation for quantifying microbial processing in deep-sea sediments (Reimers et al. 1986; Jahnke et al. 1990; Glud et al. 1994; Smith et al. 1997) only more recently has the requirement for in situ investigations of coastal sediments been acknowledged (Jahnke and Jahnke 2008; Toussaint et al. 2014).

Arguably the most important parameters for assessing the biogeochemical functioning of marine sediments are the distribution and exchange of O_2_. Benthic O_2_ availability regulates key processes in nutrient cycling, and the sediment O_2_ consumption is the most widely applied proxy for quantifying the mineralization rate of organic carbon in sediments (Glud 2008). The underlying assumption behind this approach is that most reduced constituents from the anaerobic carbon degradation are concurrently oxidized by O_2_ (Canfield et al. 1993). This may not always be entirely correct, especially in reduced sediments (Anderson et al. 1986; Therkildsen and Lomstein 1993), but in most settings and when averaged over some time the O_2_ uptake represents a robust proxy for the total benthic mineralization rate. Therefore, there have been numerous studies quantifying the benthic O_2_ exchange rate applying a range of different measuring approaches (Glud 2008 and references therein).

The in situ total O_2_ exchange (TOE) of sediments is typically quantified from the measured decline in O_2_ concentration during chamber incubations. Chamber incubations also make it possible to quantify the concurrent release of dissolved inorganic carbon (DIC) and the net benthic exchange of nutrients. The TOE represents an integrated measure of the diffusive- and the fauna-mediated O_2_ consumption of a well-defined sediment area. However, measurements of O_2_ microprofiles enable a more detailed insight on the benthic O_2_ distribution and the quantification of the diffusive-mediated O_2_ exchange (DOE). Parallel chamber incubations and microprofile measurements therefore allow quantification of the fauna-mediated O_2_ uptake (Glud et al. 2003). Latest, eddy covariance has been introduced for quantifying the benthic O_2_ exchange rate (Berg et al. 2003). The approach derives the benthic O_2_ exchange rate from concurrent measurements of the near-bed fluctuations in the O_2_ concentrations and the vertical flow velocity without disturbing the sediment or natural drivers of the solute exchange. The eddy-derived O_2_ exchange (EOE) therefore integrates the O_2_ consumption across a large (10–100 m^2^), but variable upstream area of the seabed (Berg et al. 2007). The three in situ approaches are highly complementary, and combining the approaches provides the most detailed insight on environmental factors regulating the benthic O_2_ exchange rate that can be provided.

Here we explore the benthic O_2_ dynamics of cohesive, infauna-rich, coastal sediments applying all three measuring approaches. The in situ measurements are complemented by laboratory-based investigation of O_2_ dynamics around the dominant infauna using two-dimensional planar optode technology and three-dimensional computer-aided tomography. Combined, the measurements provide an unparalleled insight into benthic O_2_ dynamics and governing factors in typical sea loch sediments. The insight is used to discuss implications for benthic carbon mineralization and nutrient turnover as inferred from concurrent in situ measurements of NO_3_^−^ microprofiles and net exchange of DIC, NO_3_^−^, NH_4_^+^, PO_4_^3−^, dissolved Si (DSi) and N_2_.

## Materials and Methods

### Study Site and Sampling Campaigns

**Fig. 1:**
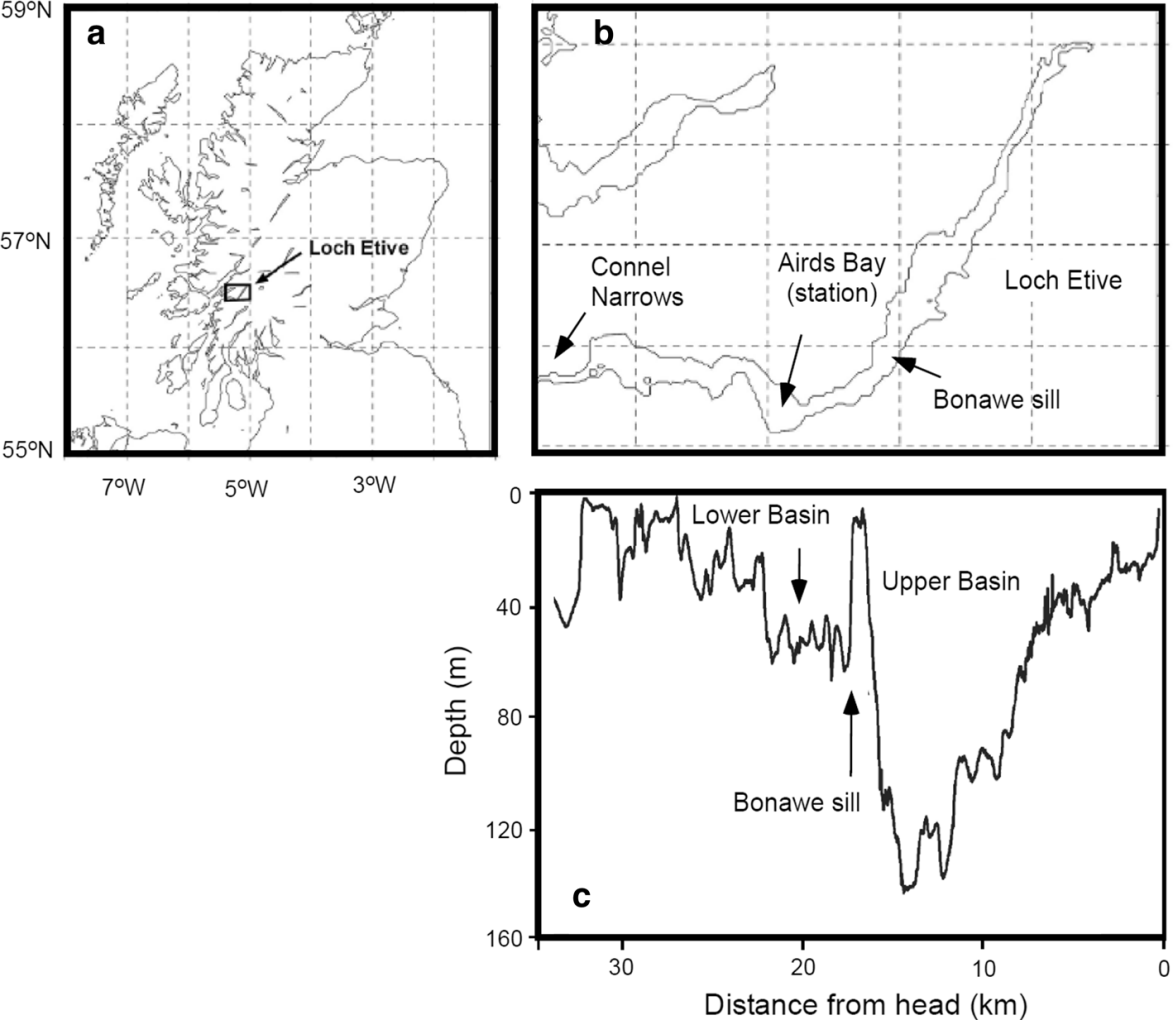
**a** Map showing the location of Loch Etive in West Scotland, UK. **b** An enlargement of Loch Etive including the locations of the major sills at Connel Narrows and Bonawe enclosing the study site in Airds Bay. **c** Bathymetry of Loch Etive along the deepest points. **a**, **b** is modified from Inoue et al. (2011), while c is modified from Overnell et al. (2002)

Loch Etive is an approximately 30-km-long glacial fjord on the west coast of Scotland (Fig. 1). Sills create a series of smaller basins, but the fjord can conveniently be divided into two major basins (Fig. 1b, c; Edwards and Edelstein 1977). The fjord has a relatively high freshwater discharge which, in combination with shallow sills at Connel Narrows and Bonawe, leads to a relatively long residence time of the saltier bottom water of the two basins (Austin and Inall 2002; Overnell et al. 2002). The present work was conducted at Airds Bay (56°27.334′N; 5°14.248′W) in the better ventilated seaward lower basin (Fig. 1). The average depth of the depth recorder of the respective in situ instruments ranged between 56 and 70 m with an overall average of 65 *±* 4 (*n* = 18) m. During the study period (29 October–14 November 2008), the bottom water temperature and salinity remained at 13 *±* 0.5 and 25 *±* 0.3 °C, respectively. Tidal-induced mixing generally ensure that the seaward basin is relatively well oxygenated, but during the study period the measured bottom water O_2_ concentration tended to decline from 181 *±* 2 to 171 *±* 3 μmol L^−1^ and the overall average value was 174 *±* 5 μmol L^−1^ (*n* = 99) corresponding to 60 % air saturation.

In total, the station was visited 11 times during the study period and on 8 occasions one or more of four instrumental tripods was deployed. The tripods were lowered from the RV *Calanus* or the RV *Soel Mara* with a buoyant rope and were moored with a surface buoy. A 30-kg weight fixed to the rope was placed 30–50 m from the instruments so that any drag from the surface buoy was not conveyed to the tripods at the sea bed. The respective tripods were deployed for periods of 6–60 h depending on weather conditions, logistical constraints and the type of instrumentation. One of the tripods always carried a conductivity, temperature, depth (CTD) instrument (XR-420, RBR) equipped with an O_2_ optode (Tengberg et al. 2006; Aanderaa) to monitor physico-chemical parameters in the bottom water.

In total we conducted 7, 6 and 5 successful deployments with a transecting microprofiler, a benthic chamber and the eddy covariance instrument, respectively. In addition, an underwater camera system (ocean imaging systems) set in time lapse imaging mode and equipped with strobe light was deployed twice to assess the sediment appearance and the dominant macrofauna in the study area. Sediment recovered by the benthic chamber was used to quantify the benthic fauna community. In addition, sediment recovered by a van Veen grab was used to collect the dominant infauna species (*Amphiura filiformi*s) for more detailed laboratory investigations on sediment oxygenation as induced by fauna activity.

Smaller subsets of data from the current study have previously been presented, with the aim of evaluating (1) implications of benthic boundary layer dynamics for the O_2_ exchange as derived by eddy covariance (Holtappels et al. 2013) and (2) procedures for assessing in situ friction velocities (Inoue et al. 2011). Here we present the entire reanalysed data set with the aim of evaluating benthic mineralization and nutrient regeneration in these highly bioturbated sediments.

### Laboratory Investigation of Fauna-Induced O_2_ Dynamics

Recovered sediment was submerged in air-flushed bottom water and kept at in situ temperature for 1 month. Hereafter the sediment was carefully transferred to narrow Perspex aquaria (*L* × *W* × *H*: 12 × 2 × 12 cm) equipped with O_2_ quenchable planar optodes (see below). During sediment transfer any observed macrofauna was manually removed while maintaining the overall sediment structure. The sediment-filled aquaria were placed in bottom water kept at in situ temperature and at 100 % air saturation. After 4 weeks, freshly sampled specimens of *A. filiformis* from the study site were introduced to the aquaria, one or two specimen per aquaria. The animals quickly dug into the sediment, and three aquaria with four animals which had positioned themselves along the planar O_2_ sensor were selected for detailed imaging, one aquarium at the time. Images with the planar optode system were obtained with a frequency of 5 min for a total of up to 30 h while the selected aquarium at each occasion was inserted into a larger thermo-regulated tank filled with bottom water from the sampling site. The overlying water was continuously flushed with an air pump to ensure constant temperature and 100 % air saturation.

The applied planar optode system used in the study was similar to previously described systems (Holst et al. 1998; Frederiksen and Glud 2006) and will only be presented briefly. The 11 × 11 cm large transparent planar optode was based on an O_2_ quenchable ruthenium(II) luminophore immobilized in polystyrene and coated onto a transparent polyester support foil. The sensor was interrogated by a fast gateable 12-bit Charged Coupled Device camera (CCD, SensiCam, PCO.de) equipped with a 60-mm Nikon macrolens and a 570-nm-longpass filter (UQGoptics.com). Excitation light was delivered from four blue high-power LEDs (LXHL-LR3C, Luxeon.com) equipped with a 470-nm-shortpass filter (UQGoptics.com). The operation of camera and LEDs was synchronized via a custom-made PC-controlled trigger box using the software Look@Molli (Holst and Grunwald 2001). The luminescent lifetime was inferred from two well-defined time frames configured by the interactive software. Recorded images were calibrated using the average luminescent lifetime of two areas of the homogenous planar optode that were exposed to known O_2_ levels (100 % air saturation in the overlying water and 0 % air saturation in deep presumed anoxic layers), using a modified rearranged Stern–Volmer equation (Glud et al. 1996):

1 $$ C = {\tfrac{{\tau_{0} - \tau }}{{K_{\text{SV}} \cdot (\tau - \alpha \tau_{0} )}}} $$

where *τ*_0_ is the luminescent lifetime in the absence of O_2_, *τ* is the lifetime in the presence of O_2_ at a level of *C*, *K*_SV_ is the Stern–Volmer quenching constant, and *α* is the non-quenchable fraction of the luminescent signal which was experientially determined to be 0.2. All images were recorded in ambient darkness.

To visualize the organization of intact burrow structures in the sediment, one Perspex aquarium (*L* × *W* × H: 16 × 8 × 16 cm) with recovered sediment and animals was kept at in situ temperature and was transported to a Toshiba Aquilion medical CT (computer-aided tomography) scanner at the local hospital (Lorn & Island Hospital, Oban, Argyll, UK). The CT scanner was operated with a power setting of 120 kV and 175 mA, and the resolution of the images obtained by the scanner was 0.26 mm × 0.26 mm × 1.00 mm (or 0.068 mm^−3^). The total scanning procedure took about 1 s. Subsequent image analyses were performed by the freeware ImageJ.

### In Situ Microprofiling Instrument

In situ microprofiles of O_2_ and NO_3_^−^ were obtained by a previously presented transecting microprofiling instrument (Glud et al. 2009a) that for the respective deployments was equipped with 2–4 Clark-type O_2_ microelectrodes (Revsbech 1989) and 2 NO_3_^−^ microelectrodes (Revsbech and Glud 2009). During one deployment the instrument was also equipped with two H_2_S microsensors (Kühl et al. 1998), but no free H_2_S could be detected in the upper 5 cm of the sediment. A titanium cylinder containing electronics and the mounted sensors was placed on a sledge that could move a total horizontal distance of 90 cm in increments of 0.7 cm obtaining one set of microprofiles at each position. The instrument could therefore obtain numerous profiles during a single deployment (Glud et al. 2009a). However, given the high density of infauna at the present study site, the microsensors were frequently damaged during the initial measurements, and we only obtained a total of 50 intact microprofiles during the 7 deployments. The O_2_ sensors had a tip diameter of 1–5 μm, 90 % response time <0.5 s and stirring sensitivity <0.5 % (Revsbech 1989; Gundersen et al. 1998), while typical NO_3_^−^ sensors had an outer diameter of ~150 μm, a 90 % response time of 1–2 min and were insensitive to stirring (Revsbech and Glud 2009). The sensors responded linearly towards solute concentrations, and the signals were calibrated against known concentrations in the bottom water and low readings in deep sediment layers that were presumed to be free of O_2_ and NO_3_^−^. The NO_3_^−^ microsensor is cross-sensitive to NO_2_^−^ and N_2_O, but as the concentration of these solutes generally is well below 1 μmol L^−1^ in marine sediments, such potential interference was ignored (Revsbech and Glud 2009). Microprofiles were measured at a vertical resolution of 0.2 mm, and sensors were kept at each measuring depth for 2 min to ensure that the NO_3_^−^ sensors had responded fully before recording the data. The diffusive solute exchange (J) was calculated from Fick’s first law of diffusion: *J* = −D d*C*/d*Z*, where *D* is the molecular diffusion coefficient and d*C*/d*Z* is the slope of the linear concentration gradient. When possible, we applied the concentration gradient of the diffusive boundary layer (DBL) and the molecular diffusion coefficient at in situ temperature and salinity (*D*_0_ = 1.63 10^−5^ cm s^−1^) (Rasmussen and Jørgensen 1992). However, in some instances the spatial resolutions in the DBL was insufficient for applying this procedure and in other instances we wanted to calculate fluxes below the sediment surface. For instance, this was the case when deriving the nitrification rate in the oxic surface layer, where the upward and downward flux from the concentration peak was calculated, the first representing an efflux to the overlying water and the latter sustaining underlying denitrification (Glud et al. 2009b). In those instances, we applied the concentration gradient in the sediment and the tortuosity corrected molecular diffusion coefficient as derived from *D*_*s*_ = *D*_0_ *φ* ^(*m*−1)^, where *φ* is the porosity and *m* was a sediment constant assumed to be 3 (Ullmann and Aller 1982). Both procedures were tested on a few profiles which provided very similar results for the diffusive O_2_ exchange across the sediment water interface (not shown).

### In Situ Chamber Incubations

To quantify the total benthic exchange rates of O_2_, N_2_ and nutrients, we deployed the benthic chamber lander Elinor (Glud et al. 1995) which is based on the design of the BECI-lander (Jahnke and Christensen 1989). The squared chamber enclosed ~900 cm^2^ of sediment with an overlying water column of 10–15 cm. As the tripod settled at the seabed, the chamber was inserted in the sediment and after 1 h the incubation was initiated by lid closure. During incubations a central cross-bar stirred the overlying water at 10 RPM (revolutions per minutes) which ensured a well-mixed overlying water column and an average diffusive boundary layer (DBL) thickness on the order of ~0.5 mm (Glud et al. 1995). During incubations, the O_2_ concentration of the enclosed water was measured every 30 min using a commercially available O_2_ optode (AADI 4835, Andreaa) (Tengberg et al. 2006, Andreaa; AADI 4835), and data were internally stored. Additionally, 5–8 water samples were taken from the enclosed water at regular intervals by spring-loaded, 50-mL glass syringes that were triggered by a motorized spindle controlled by the preprogrammed electronics (Glud et al. 1995). The sampled water was replaced via a 3-mm (inner diameter), 50-cm-long, coiled tube connecting the chamber to the outside water. At the end of the incubation, a scoop closed beneath the chamber and the incubated sediment core was recovered with the tripod (Jahnke and Christensen 1989). Upon recovery, each of the 50 mL water samples were divided into three subsamples and preserved for subsequent concentration measurements of dissolved inorganic carbon (DIC), N_2_ and nutrients (NH_4_^+^, NO_3_^−^, PO_4_^3−^, DSi). For DIC, 12 mL of subsample was stored in gastight Excitainers (Labco, UK) spiked with 200 μL saturated HgCl_2_ until analysis on a coulometer (CM5012 UIC Coulometrics; precision *±* 2 μmol L^−1^) (Johnson et al. 1987). Samples for N_2_–Ar measurements were stored at 15°C in submerged 7-mL glass stoppered vials with 100 μL saturated HgCl_2_ until analysis on membrane inlet mass spectrometer (Balzers QMS422; precision for N_2_ determination *±* 0.5 μmol L^−1^) (Eyre et al. 2002). For nutrients, 25 mL of water was filtered through 25-mm, 0.45-μm-pore-size filters (Whatman GF/F) and either frozen at −18 °C (for NH_4_^+^, NO_3_^−^, PO_4_^3−^) or stored at 4 °C (for DSi) until analyses. The samples were analysed within 1 week after sampling using a LACHAT QuikChem 8500 Flow Injection AutoAnalyser (LaChat Instruments) and corrected for blanks (Sargasso Seawater from OSIL). The precisions as assessed by replicate measurements of standards in the relevant measuring range (*n* = 6) were 0.03, 0.07, 0.01 and 0.2 μmol L^−1^ for NH_4_^+^, NO_3_^−^, PO_4_^3−^ and DSi, respectively. The total exchange rate of the respective solutes was quantified assuming linear development in concentration changes during incubations and by accounting for the enclosed volume of water.

### Eddy Covariance Measurements

The applied aquatic eddy covariance (AEC) system was similar to the one described by Berg and Huettel (2008). The main components consisted of an acoustic Doppler velocimeter (ADV, Vector, Nortek) and an O_2_ microelectrode with the same measuring characteristics as described above. The O_2_ microsensor signal was relayed to the ADV via a submersible amplifier (McGinnis et al. 2011). The vertically aligned ADV recorded the longitudinal, traverse and vertical velocity components along with the output from the O_2_ microsensor at a frequency of 32 Hz, which allowed full resolution of the current-driven turbulence transporting O_2_ to the seafloor. The data were recorded in 15-min-long bursts consisting of 14.5 min of continuous sampling followed by 0.5-min sleep period with no data collection. The ADV sampling volume (1.5 × 1.5 cm) was located ~10–15 cm above the seabed surface, and the O_2_ microelectode tip was positioned a few mm outside the sampling volume of the ADV to ensure uncompromised velocity measurements. Prior to deployment, the AEC O_2_ microsensors were left to polarize for a minimum of ~12 h to minimize sensor drift and their “zero readings” were measured by dipping the sensor tips in a sodium dithionite solution. Before flux extraction, each sensor was calibrated against the known bottom water concentration of O_2_ (see above) and its zero reading.

O_2_ exchange rates, one for each 15-min burst, were extracted from the raw EC data set using the software package EddyFlux version 3.00 (P. Berg unpubl.). In short, the raw EC data were averaged down to 8 Hz which provided sufficient resolution to describe the entire frequency spectrum carrying the flux signal. Spectral analysis was applied to selected data sequences to validate this assumption. Abnormalities in the EC data record, usually caused by debris colliding with or attaching to the O_2_ sensor, were then removed as described by Lorrai et al. (2010), Hume et al. (2011) and Berg et al. (2013). In total ~18 % of the data were discarded by this procedure. To compensate for any sensor tilt, the velocity field for each burst was rotated which nullified the transverse and vertical mean velocities. To compensate for the limited response time of the O_2_ microelectrode (<0.5 s) and the physical separation between the O_2_ sensor tip and the centre of ADV’s measurement volume, a time lag correction was applied to each burst. This was done by repeating the flux calculation for each burst while shifting the 8-Hz concentration data in 1/8 s increments until the numerically largest flux was found (McGinnis et al. 2008; Lorrai et al. 2010). The identified time shifts, one for each burst, varied with the mean current velocity and its direction, but averaged over all deployments, this correction increased the flux by 25 % (McGinnis et al. 2008; Lorrai et al. 2010; Donis et al. 2015). The O_2_ fluxes, in mmol m^−2^ day^−1^, were then extracted from the vertical ADV velocity (*w*) and the measured O_2_ concentrations (*C*) as the covariance $w^{{\prime }} C^{{\prime }}$, where the fluctuation components, $w^{{\prime }}$ and $C^{{\prime }}$, are deviations from a least-squares linear fit to the data in each burst (Lee et al. 2004; Berg et al. 2009). The variation in the derived EOE was quantified by both the SD and standard error of the burst values during the respective deployments.

Recently, it has been shown that stirring sensitivities of O_2_ microelectrodes can confound the eddy-derived O_2_ exchange EOE and provide an artificial flux contribution (Holtappels et al. 2015). Provided the sensor characteristics and measuring conditions the maximum effect was assessed by the procedure described in Berg et al. (2015) and amounted to 8 % of the measured flux. However, as the applied sensors will not express maximum stirring sensitivity from all current directions (Holtappels et al. 2015) the average effect for the respective deployments must have been considerably less. In the following, we have therefore ignored this minor offset of the derived EOE.

### Basic Sediment Characteristics and Quantification of Macrofauna

Sediment porosity and organic content were determined in two sediment cores that were subsampled from a box corer that had been recovered with a clear overlying water phase. The cores were sectioned into 1-cm slices down to 10 cm depth, and porosity was quantified from the weight loss after drying for 24 h at 105 °C and the wet density. The organic content of the dried sediment was determined as the relative weight loss upon ignition after 24 h at 450 °C without an acidification step. However, relevant sedimentary carbonates remain stable at this temperature and should not confound the assessment of the organic content (Krom and Berner 1983). For quantification of macrofauna, four intact sediment cores (900 cm^2^) that were recovered by the benthic chamber lander were sieved through a 1-mm mesh, and animals were collected and stored in 70 % ethanol for subsequent taxonomic determination.

## Results

### Basic Sediment Characteristics and Fauna-Induced Oxygenation

**Fig. 2:**
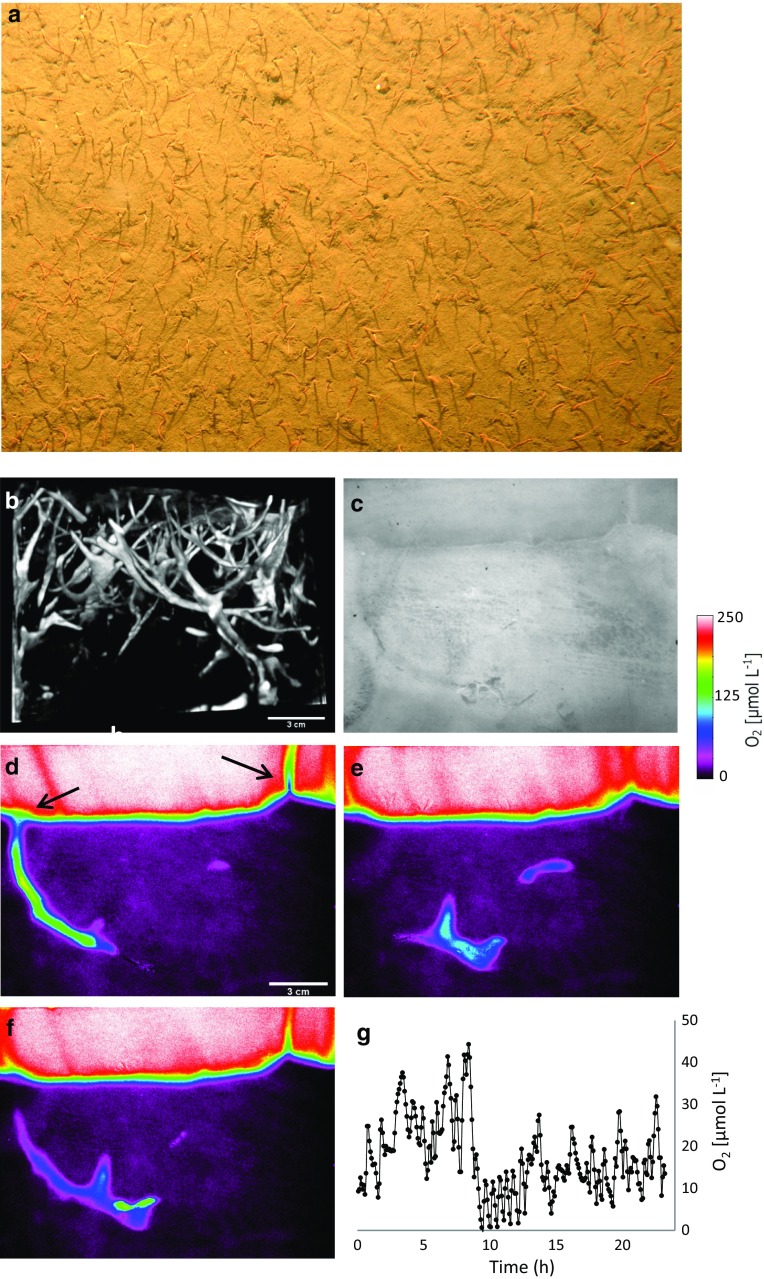
**a** One typical in situ image (89 × 63 cm) of the seabed at the study site showing a high density of arms from *A. filiformis* that are extending from the sediment surface. **b** A 3D, CT scan of burrow structures in an intact block of sediment containing actively ventilating brittle stars at natural densities. **c** One typical black-and-white image selected from a 30-h time series of images at the sediment water interface obtained at a frequency of 5 min. The image was obtained through a transparent O_2_ optode, and the central cavity with the disc and arms extending to the left and right from a single specimen of *A. filiformis* can be observed. **d**–**f** depict three selected images of the O_2_ distribution within and around the ventilated burrow system. One arm is used to channel air-saturated water into the central cavity while O_2_-depleted water is exhaled to the right (indicated by arrows). **g** The O_2_ concentration within the central cavity as extracted from 25 h of continuous O_2_ images. The full movie on animal activity in black-and-white and the concurrent O_2_ dynamics is available in the supplementary material

The cohesive sediment consisted of silty mud, and the surface appeared well mixed as the upper 5 cm exhibited no distinct vertical profiles in porosity or organic content (not shown). On average, the surface sediment had a porosity of 0.90 *±* 0.05 (*n* = 10), while the dry organic content was 12.6 *±* 1.2 % (*n* = 10). This is a relatively high value for coastal sediments in general, but it aligns well with previous measurements in Loch Etive and other Lochs in the region (Overnell et al. 1996). Below the 5-cm surface layer, both porosity and organic content tended to decline to minimum values of 0.88 and 10.5 % at the deepest sampling point of 10 cm (not shown). The sediment hosted abundant macrofauna with a total wet-weight biomass of 272 *±* 84 g m^−2^ (*n* = 4) as assessed from intact sediment cores of the benthic chamber. The faunal abundance was clearly dominated by ophiuroids (1070 *±* 280 m^−2^), followed by polychaetes (370 *±* 223 m^−2^) and small bivalves (330 *±* 16 m^−2^). These taxonomic groups accounted for >95 % of the abundance, and the single most abundant species was *A. filiformis* accounting for 820 *±* 139 m^−2^. In situ time lapse images of the sediment surface clearly revealed the dense coverage of arms of *A. filiformis* extending from the sediment and filter feeding in the bottom water (Fig. 2a). A three-dimensional CT scan of an intact sediment block showed how the sediment was interwoven by burrows of *A. filiformis.* Typically, the animals were sitting slanted in a central cavity with 2 or 3 arms forming open channels to the sediment surface (Fig. 2b). We only scanned one intact sediment block, and for this sample, the density of *A. Filiformis* burrows amounted to 1503 m^−2^ and the overall burrow volume in the upper 10 cm was 4824 cm^3^ m^−2^. This equates to 4.8 % of the sediment volume of the upper 10 cm. The O_2_ images of intact burrow systems documented how filter feeding animals drew oxygenated water down along one arm and into the central cavity while O_2_ depleted, and on some occasions even anoxic, water left through another outlet (Fig. 2d, f). Typically, 2–3 of the five arms were used for irrigation, while the remaining arms moved around in (or below) the central cavity. Two full 25-h-long movies showing the O_2_ dynamics and animal activity associated with an inhabited burrow can be seen in the supplementary material. Generally, the irrigation was very dynamic and in most cases without any distinct rhythm. This resulted in a highly variable O_2_ concentration within the burrow system and the central cavity (Fig. 2d–f). However, while the arm channels frequently experienced complete anoxia, the central cavity generally remained oxic with values ranging between 1 and 50 μmol L^−1^ (Fig. 2g). Addition of food particles (Interpet Liquifry Marine) to the aquaria clearly stimulated the ventilation activity of the animals (not shown). *A. filiformis* therefore irrigated the upper ~10 cm of the sediment with bottom water leading to a highly dynamic oxic environment in arm channels, the cavities and the surrounding sediment. Both the laboratory observations and the in situ time lapse images of the surface sediment (Fig. 2a) suggested that the animals and their burrow systems were stationary.

### In Situ Microprofile Measurements

**Fig. 3:**
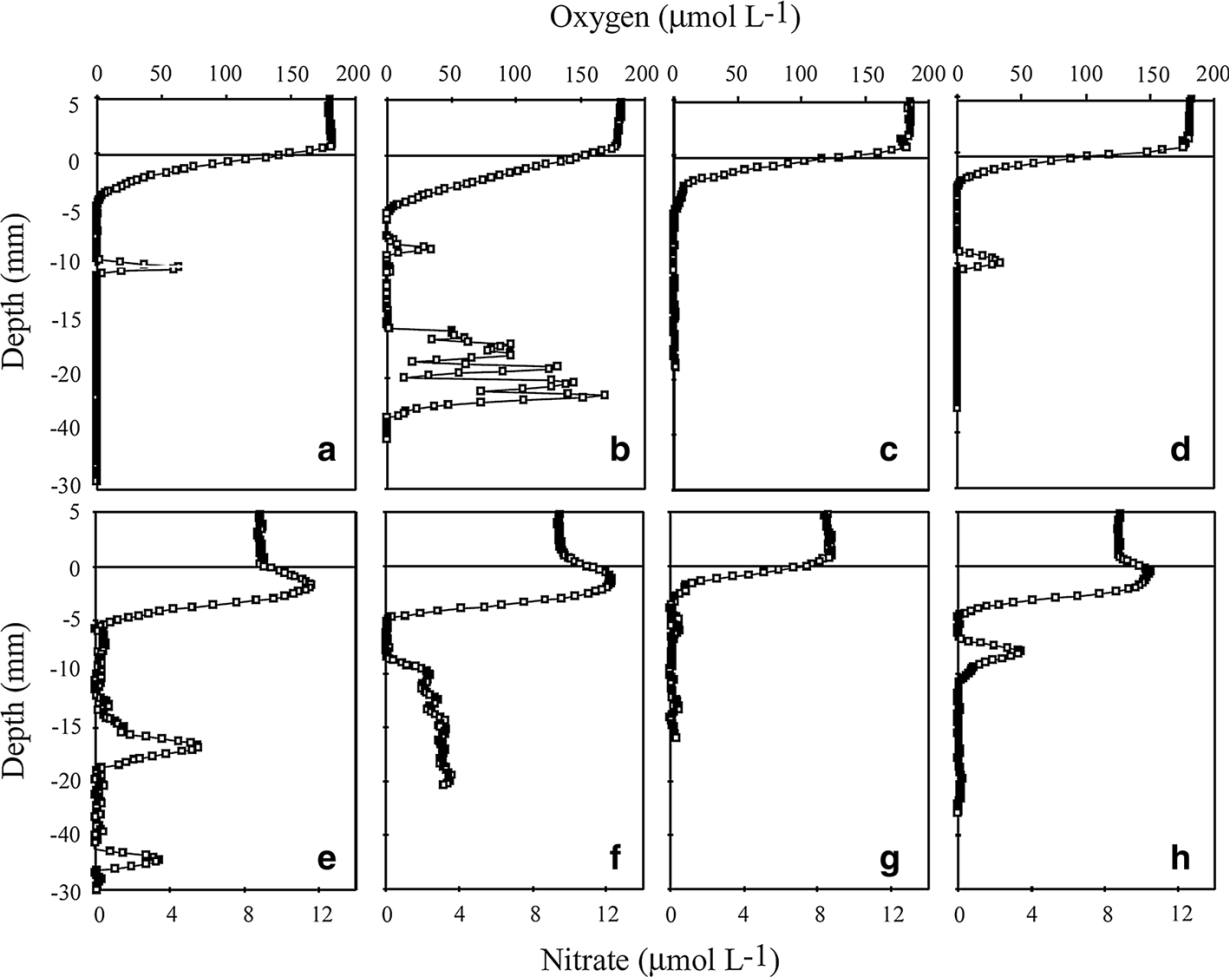
**a**–**d** Typical O_2_ microprofiles as measured by the transecting profiling instrument. The estimated sediment surface as reflected by a slight shift in the concentration slope is indicated by the *thin horizontal line*. Many profiles showed clear signs of irrigation with O_2_ peaks deep within the sediment presumably penetrating irrigated burrows (**a**, **d**) or larger cavities (**b**). **e**, **f** depict typical microprofiles of NO_3_^−^ distribution at the sediment–water interface. Some profiles reveal intense NO_3_^−^ production (nitrification) at the oxic surface (**e**–**h**). As for O_2_, irrigation clearly transported NO_3_^−^ deep into burrows (**e**, **h**) or larger cavities (**f**) of the sediment

During the seven deployments of the profiling instrument, we successfully obtained 36 O_2_ and 14 NO_3_^−^ microprofiles (and a few H_2_S microprofiles documenting that the upper 5 cm of the sediment was H_2_S-free). Most microsensors broke during profiling, presumably as a consequence of the high density of infauna. On average ~50 % of the microprofiles were visually affected by burrow structures and irrigation, as reflected by subsurface concentration peaks and irregular signals (Fig. 3). In most cases these anomalies were encountered in the deeper otherwise anoxic sediment layers well below the oxygenated surface layer (Fig. 3). Only including the apparently undisturbed surface layer, the microprofiles still exhibited an extensive small-scale variations (Fig. 3), and the average O_2_ penetration depth along the sediment surface was 4.0 *±* 1.3 mm (*n* = 36). Only including O_2_ measurements that at the surface appeared unaffected by animal activity provided an average diffusive O_2_ exchange (DOE) of −7.5 *±* 2.3 mmol m^−2^ day^−1^ (*n* = 26). The measured NO_3_^−^ microprofiles also exhibited an extensive small-scale variation; while most profiles had clear nitrification peaks at the sediment surface and a relatively deep NO_3_^−^ penetration (Fig. 3e, f, h), other profiles reflected a fast exhaustion of NO_3_^−^ just below the sediment surface (Fig. 3g). Consequently, NO_3_^−^ penetration depth at the apparently undisturbed sediment–water interface varied by a factor of ~5, but on average amounted to 6.2 *±* 3.5 mm (*n* = 14). Correspondingly, the diffusive net exchange of nitrate at the sediment water interface varied extensively, but on average the exchange rate across the sediment water interface amounted to 0.12 *±* 0.40 mmol m^−2^ day^−1^ (*n* = 14). As for O_2_, the irrigation by *A. filiformis* clearly infused NO_3_^−^ enriched water deep into the burrow structure well below the primary interface, and the irrigation enhanced the overall net exchange of solutes (Fig. 3).

### Benthic Chamber Incubations

**Fig. 4:**
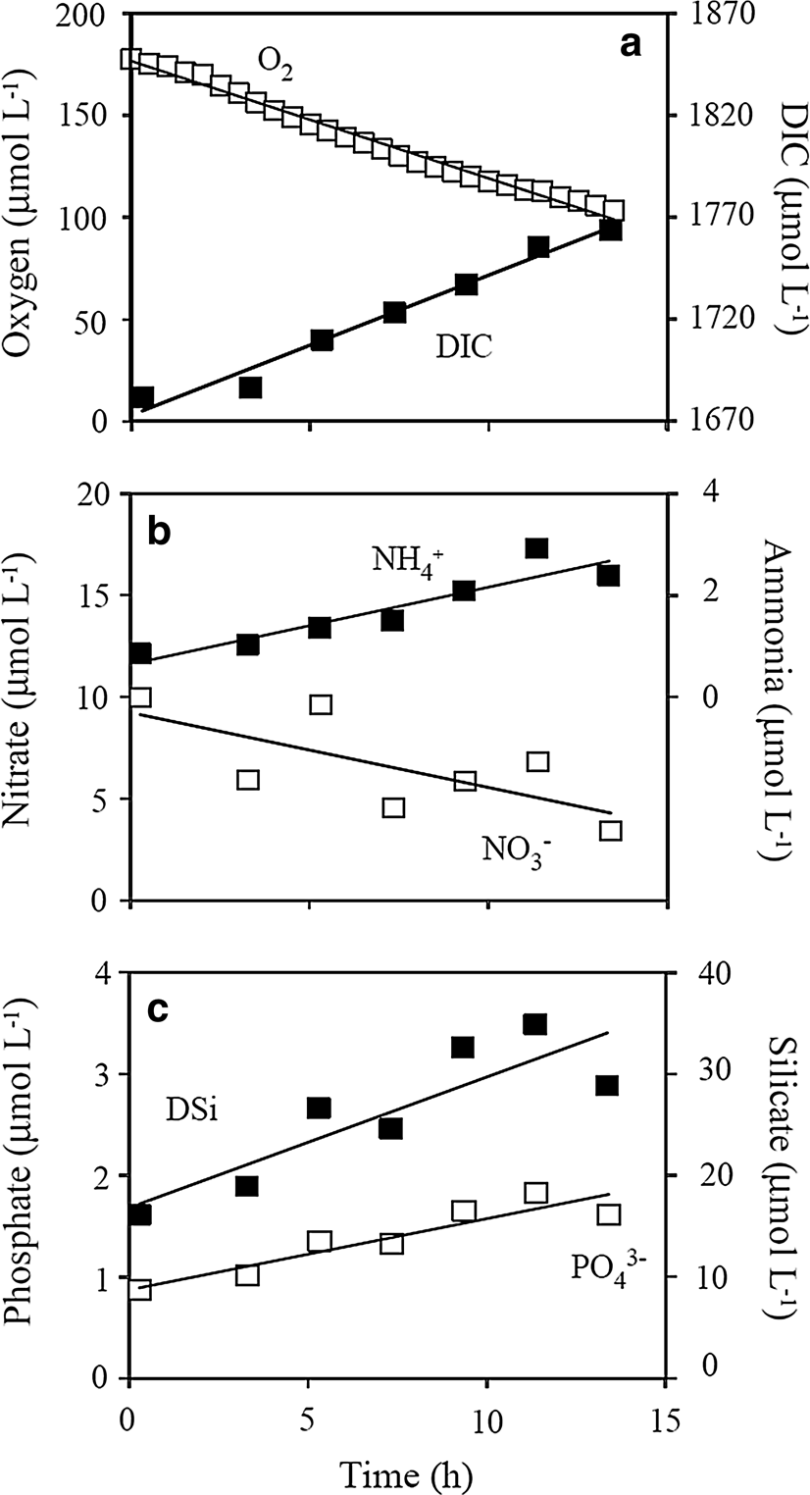
Changes in O_2_, DIC and nutrient concentrations within a single in situ chamber incubation conducted at 5 November 2008 at 65 m water depth (deployment 6). Flux rates were derived from linear approximations of the concentration changes and by accounting for the enclosed volume of water

**Fig. 5:**
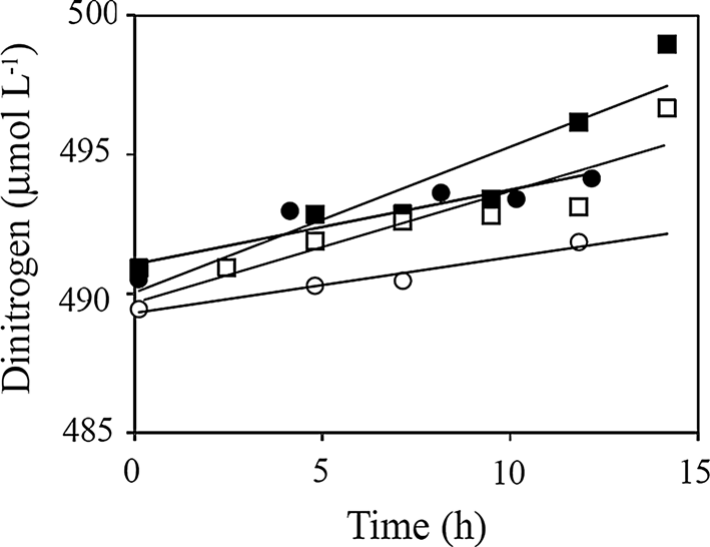
Changes in N_2_ within four separate in situ chamber incubations as derived from overall changes in the measured N_2_–Ar ratio. Samples containing small bubbles after sampling or storage were discarded. Flux rates were derived from linear approximations of the concentration changes accounting for the enclosed volume of water

Whereas the microprofiles resolved an extensive small-scale variation in distribution and diffusive exchange rates of O_2_ and NO_3_^−^, the benthic chamber incubations provide an integrated total net-exchange rate across areas of ~900 cm^2^. In most cases, the concentration changes within the enclosed water volume could be reasonably well approached by a simple linear relation (Fig. 4). In total we conducted 6 successful chamber deployments, but DSi analyses were only carried out on the last two deployments. Overall total O_2_ exchange (TOE) rates amounted to −14.9 *±* 2.5 mmol m^−2^ day^−1^, while the DIC release amounted to 15.5 *±* 3.4 (Table 1) providing an average sediment respiratory quotient (RQ) of 1.03 *±* 0.11. The sediment consistently effluxed NH_4_^+^, PO_4_^3−^ and DSi, while there was a net uptake of NO_3_^−^ that scaled with the efflux of NH_4_^+^ (Table 1). On four occasions we managed to obtain reliable samples for N_2_ determination (Fig. 5), and they consistently documented a net efflux of N_2_ (Table 1).

**Table 1:** Total solute exchange rates (mmol m^−2^ day^−1^) as derived by in situ chamber incubations

Deployment	O_2_	DIC*	NO_3_^−^	NH_4_^+^	PO_4_^3−^	DSi**	N_2_***
1	−16.8	19.0	−0.03	0.41	0.83	–	1.67
2	−16.6	19.0	−1.00	0.18	0.40	–	0.61
3	−14.1	12.8	−0.21	0.21	0.08	–	1.44
4	−10.4	11.2	−0.12	0.85	0.12	–	0.38
6	−16.8	17.2	−1.05	0.43	0.20	3.60	–
7	−14.9	13.5	−0.20	0.30	0.13	2.98	–
Average	−14.9 ± 2.5	15.5 ± 3.4	−0.43 ± 0.42	0.40 ± 0.22	0.29 ± 0.26	3.29 ± 0.31	1.02 ± 0.54

* Dissolved inorganic carbon

** Dissolved silicate

*** Note that for converting the N_2_ flux into N equivalents this value must be multiplied by 2

### Benthic O_2_ Consumption as Quantified by Eddy Covariance

The five deployments of our eddy covariance instrument provided a total of 119 h of successful measurements. While the benthic chamber incubations integrate the microscale variability resolved by the microprofiles, the eddy O_2_ exchange (EOE) non-invasively resolves the net O_2_ exchange across a large (10–100 m^2^), area upstream from the measuring point. The approach therefore overcomes potential mesoscale variations in O_2_ consumption at the seabed. The area included in the EOE measurement changes with the current direction. However, in the present study there was no apparent relation between current direction and the size of the resolved fluxes, indicating a homogenous average O_2_ consumption at the scale of the footprint size. In situ images of the sea floor in the area also appeared similar.

**Fig. 6:**
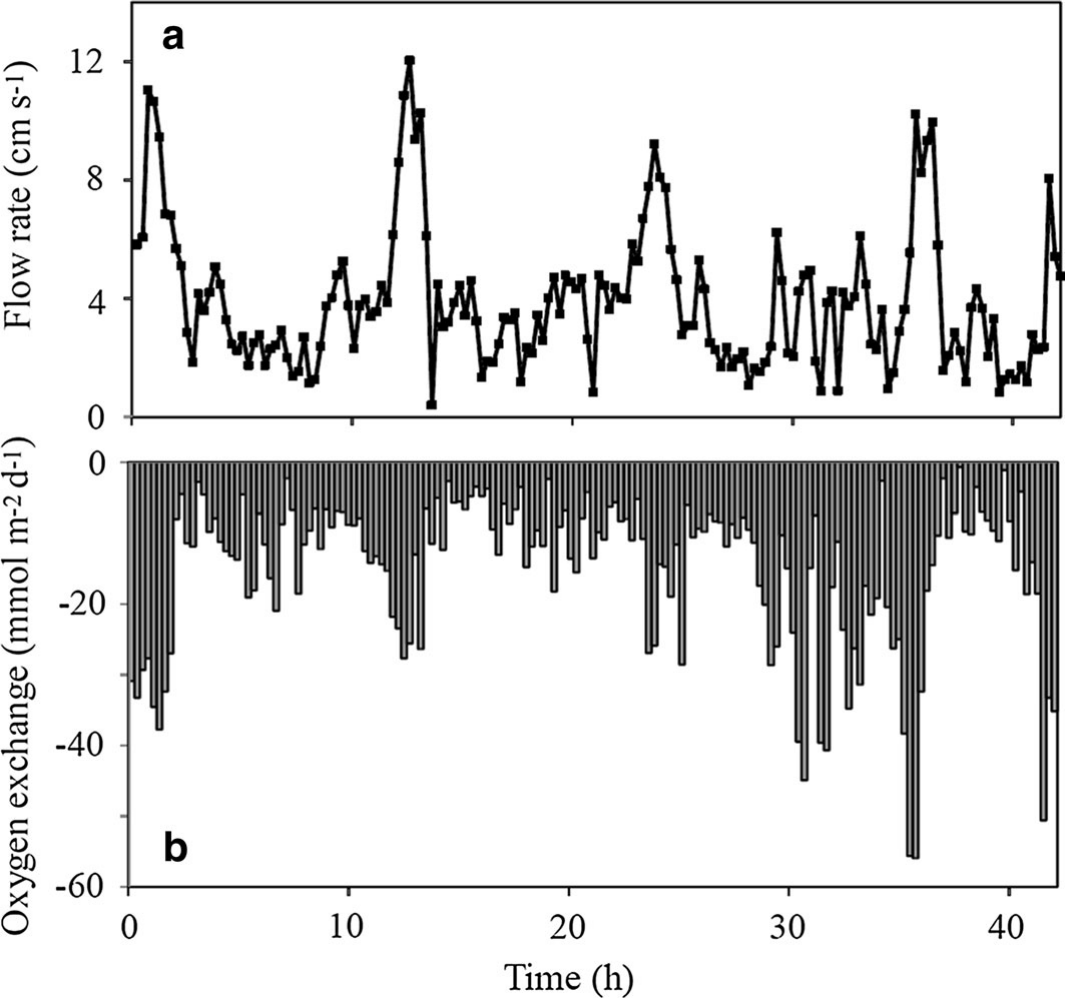
**a** Flow rates as derived by the horizontal flow components measured by the ADV of the eddy tripod during a 42-h-long deployment. Values are averages of 14.5 min of recording. **b** The concurrent O_2_ exchange rates as derived by eddy covariance in bursts of 14.5 min. The example represents a deployment from 5 to 6 November 2008 at 68 m, and a parallel data set from 7 to 10 November 2008 at 55 m has been presented previously (Holtappels et al. 2013)

The measuring approach resolved the turbulent-driven solute exchange well above the viscous sublayer, and as expected, the time series exhibited an extensive burst to burst (15-min periods) variation (Fig. 6). However, overall burst values tended to be related to the ambient flow velocities with increasing O_2_ exchange during periods of elevated horizontal flow rates driven by tides (Fig. 6). The average EOE for the respective five deployments varied between 9.5 and 15.0 mmol m^−2^ day^−1^ with an overall weighted average for the five deployments of 13.1 *±* 9.0 mmol m^−2^ day^−1^ (SE = 0.5; *n* = 493).

## Discussion

### Benthic O_2_ Uptake as Quantified by Three Different In Situ Approaches

**Fig. 7:**
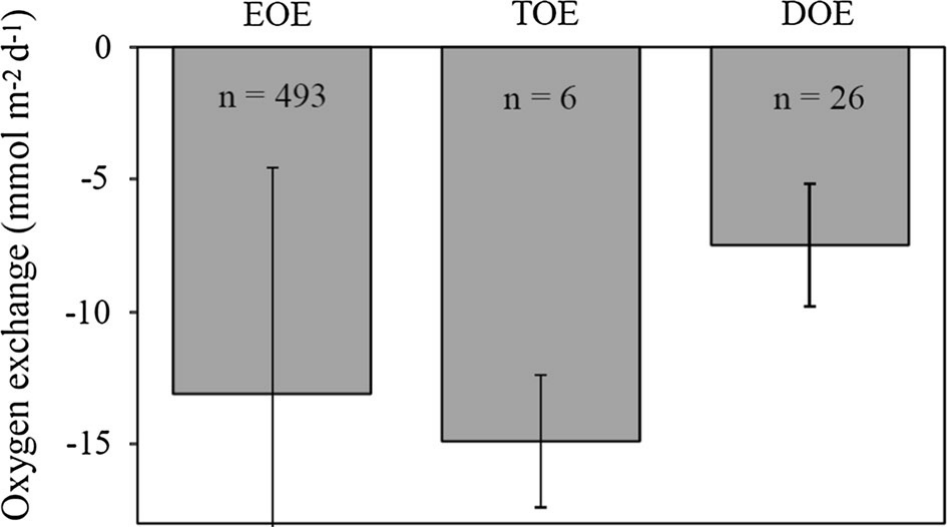
Benthic O_2_ exchange as resolved by three different in situ approaches. The error bars represent the standard deviation (For EOE the SE amounted to 0.5 mmol m^−2^ day^−1^)

We applied three in situ approaches to quantify benthic O_2_ consumption during a total of 18 deployments at an average water depth of ~65 m in Airds Bay, Loch Etive. The average O_2_ exchange rates resolved by the chamber incubations and the eddy covariance approach were similar (Fig. 7). This provides confidence in both measuring approaches. It also implies that the chamber incubations—on average—captured the relevant spatial variability and imposed hydrodynamics and a DBL thickness that maintain a near-natural solute exchange of the targeted sediments during incubations. A recent compilation of concurrent eddy deployments and chamber incubations also concluded that while TOE and EOE provide different rates in complex benthic habitats like merl beds, reefs, seagrass meadows and permeable sand—presumably due to the invasive approach of chamber incubations—values obtained in cohesive mud without conspicuous megafauna are generally similar (Attard et al. 2015 and references therein). This is further validated by the very extensive data set of the current study despite being performed in sediments with a high infauna biomass and irrigation activity.

The non-invasive EOE measurements exhibited a flow-dependent O_2_ exchange rate. This is attributed partly to the principle of the measuring technique and is partly coursed by actual sediment responses. The eddy covariance approach monitors conditions at some distance from the seabed, typically 10–15 cm, and increasing flow rates will lead to changes in the flow and the O_2_ concentration profile through the benthic boundary layer (BBL). Increasing flow will enhance downward mixing and the O_2_ concentration in the BBL and inducing a transient storage of O_2_ in the water volume below the sensors (Holtappels et al. 2013). Therefore, EOE will transiently increase with increasing flow rates as induced by the tidal forcing, and correspondingly decreasing flow rates will lead to decreasing EOE values. However, the overall long-term average value of EOE as measured over a complete tidal cycle will remain unaffected, but the EOE will exhibit variations reflecting this dynamics in the BBL. Similarly, inflow of O_2_-enriched (or depleted) water will lead to a transient increase (or decrease) in the EOE value that solely is related to equilibration in the BBL. However, O_2_ concentration in the bottom water in Airds Bay remained relatively constant during the entire study period. Using data from a different deployment in Airds Bay, than the one depicted in Fig. 6, the effect of non-steady-state distribution of O_2_ in BBL for EOE has previously been evaluated in detail (Holtappels et al. 2013). Here it was concluded that this effect indeed could explain some of the observed flow dependence of the EOE, but that other factors as well must have contributed to the observed dynamic in EOE (Holtappels et al. 2013).

Increasing flow rates have been shown to enhance the passive flushing of burrows in the sea bed (Stieglizz et al. 2000; Munksby et al. 2002). Given the extensive volume of burrows containing O_2_-depleted water, enhanced flushing as driven by increasing ambient flow rates could have contributed to the observation of flow-dependent O_2_ exchange. Also, *A. filiformis* can shift between deposit and suspension feeding (Woodley 1975) and increasing ambient flow has been shown to enhance their suspension feeding (Loo et al. 1996). It is therefore likely that changing ambient flow rates affected the in situ feeding activity and the feeding behaviour of *A. filiformis* and therefore stimulated the net uptake of O_2_. We tried to evaluate this from the in situ time lapse images, but given the image quality, it was difficult to assess a pattern in the feeding activity. Furthermore, it has also been demonstrated that increasing flow rates will compress the thickness of the diffusive boundary layer, which again will lead to a transient increase in the DOE (Jørgensen and Des Marais 1992; Glud et al. 2007). Assuming depth-independent O_2_ consumption rates in the sediment, the DOE following complete elimination of the DBL will increase by a factor of (*C*_*w*_/*C*_0_)^0.5^, where *C*_*w*_ and *C*_0_ are the O_2_ concentrations in the mixed water phase and at the sediment surface, respectively (Boudreau and Guinasso 1982). Using the O_2_ microprofiles measured in Airds Bay, the value equates to a maximum of 30 %. The flow-dependent benthic O_2_ uptake as resolved by the non-invasive EOE measurements can therefore be attributed to a combination of several effects. The presented data document that if detailed and careful data analyses are undertaken, the eddy covariance approach can provide high-quality measurements of benthic O_2_ exchange. But it is also clear that to obtain reliable average values, deployments need to be long term capturing the natural variability and the stochastic nature of turbulent-driven solute exchange.

As expected, the DOE was significantly lower than the TOE and EOE (Fig. 7. The difference between DOE and TOE is frequently used as a measure of the fauna-mediated O_2_ exchange (FOE), and for coastal settings, FOE typically accounts for ~40–50 % of the TOE (Forster et al. 1999; Archer and Devol 1992; Jahnke 2001; Glud et al. 2003; Berelson et al. 2013). The FOE of Airds Bay is estimated to equal 7.4 mmol m^−2^ day^−1^ (or 5.6 if we scale to the average EOE) being equivalent to ~50 % of the TOE and reflect the relative high biomass and irrigation activity of the infauna. A recent pulse chase experiment with ^13^C-labelled phytodetritus added to a series of sediment cores recovered from Airds Bay (water depth 70 m), identified ophiuroids as key players for processing the supplied material (Woulds et al. 2016). While ~35 % of the processed material was respired ~60 % was taken up by ophiuroids over the 6-day-long incubation. This highlights the importance of the infauna for the initial processing of settled organic material. A very detailed laboratory investigation has previously assessed the importance of *A. filiformis* for benthic O_2_ dynamics in coastal sediments (Vopel et al. 2003). The authors conducted the experiments in sediment with an average O_2_ penetration of 4.0 mm and a DOU of 8.6 mmol m^−2^ day^−1^, both values being very similar to our in situ measurements in Airds Bay. The respiration rate of adult individuals was measured to be 191 nmol O_2_ h^−1^, while microcosm incubation assessed the burrow-specific O_2_ consumption rate to be 391 nmol O_2_ h^−1^ (Vopel et al. 2003). Scaling this to the average abundance of *A. filiformis* encountered in Airds Bay (820 *±* 139 m^−2^), these animals would be responsible for an O_2_ uptake in the order of 11.5 mmol m^−2^ day^−1^. This is somewhat higher than our total estimate for FOE (7.4 mmol m^−2^ day^−1^). However, considering that the laboratory experiments were performed in normoxic water that was 4 °C warmer than the current in situ settings and the potential stress associated with laboratory conditions, the values agree within expected bounds.

The combined data show how the infauna, dominated by *A. filiformis*, markedly enhanced the benthic O_2_ uptake mainly through the dynamic irrigation of numerous complex burrows in the surface sediment. This induced highly dynamic redox conditions in the burrow environment, which must have significant implications for benthic carbon mineralization and nutrient regeneration in Airds Bay.

### Benthic Carbon and Nutrient Turnover in a Highly Irrigated Coastal Sediment

A global compilation of available in situ TOE measurements predicts an average benthic O_2_ consumption rate of 13.2 mmol m^−2^ day^−1^ for sediments at water depth of 65 m (Glud 2008). This value is bracketed by our measurements in Airds Bay (TOE: 14.9 mmol m^−2^ day^−1^ and EOE: 13.1 mmol m^−2^ day^−1^) that also are intermediary to the wide range of previously published values from sea lochs of similar water depths in West Scotland (6–28 mmol m^−2^ day^−1^)^1^ (Overnell et al. 1996; Loh et al. 2008a; Nickell et al. 2003; Cathalot et al. 2012). The average RQ as inferred from the concurrent exchange rates of O_2_ and DIC is close to unity (1.03 *±* 0.11). This implies that, on a scale of the chamber enclosed area (900 cm^2^), the reduced equivalents that are being released during anaerobic degradation are being balanced by a concurrent oxidation by O_2_, and that O_2_ consumption is a good proxy for the total benthic carbon mineralization rate, also in these sediments.

The benthic mineralization in Scottish lochs is being sustained partly by local pelagic primary production and partly by terrestrially produced material that is supplied by riverine run-off (Loh et al. 2008b, 2010). To our knowledge, there are no recent assessments of primary production or sedimentation for Loch Etive. But older seasonal studies in Airds Bay estimated an annual mesotrophic gross primary production of 70 g C m^−2^ year^−1^ (16.0 mmol C m^−2^ day^−1^) (Wood et al. 1973) and an annual sedimentation rate of 247 g C m^2^ year^−1^ (56.4 mmol C m^−2^ day^−1^) (Ansell 1974), suggesting substantial supply of terrestrial material and sediment focusing in the basin.

The measured organic content at 10 cm sediment depth equates to an organic carbon (OC) content of 4.2 % dry weight assuming that 40 % of the organic matter consists of carbon. This aligns well with previous OC measurements of 4.9 % in surface sediments of Airds Bay (Loh et al. 2008b). The sedimentation rate in central Airds Bay has been assessed previously using a profile of excess ^210^Pb and estimated to equal 0.59 cm year^−1^ (Overnell 2002). Using this information and applying the measured porosity (0.88 vol:vol) and sediment density (2.7 g cm^−3^) at 10 cm sediment depth, the annual carbon burial rate amounts to 95 g m^−2^ year^−1^ (21.4 mmol m^−2^ day^−1^). Our benthic mineralization carbon rates were obtained in autumn and cannot be directly extrapolated across seasons. However, acknowledging seasonal (and interannual) variation in the production and the supply of organic material, adding our measurement of benthic carbon mineralization (~15 mmol m^−2^ day^−1^; Table 1) and the derived carbon preservation rate gives a total sedimentation rate of 161 g C m^−2^ year^−1^ which scale to the estimated sedimentation rate of Ansell (1974). Based on these figures we can calculate a carbon burial efficiency of ~59 % which is consistent with the high sedimentation rate and substantial terrestrial inputs (Canfield 1994; Ståhl et al. 2004; Blair and Aller 2012).

Detailed investigation of sedimentary δ^13^C values and lignin content along transects in Loch Etive (and other sea lochs in the area) concluded that the terrestrial material contributed significantly to the sediment inventory of organic material (Loh et al. 2002, 2008a, b). The authors also identified a fraction of terrestrial material that was highly labile with low C–N ratios and that this fraction apparently was efficiently degraded—especially in the central Airds Bay (Loh et al. 2008a). Furthermore, the rate constant for the degradation of refractory lignin was highly elevated in Airds Bay (Loh et al. 2008a). These observations and the fact that the benthic O_2_ consumption exceeds the local primary production strongly suggest that terrestrial carbon supply was important in sustaining the benthic community and that the turnover of refractory organic material was stimulated in these sediments. Sediment reworking and dynamic redox conditions have been shown to stimulate degradation of organic material (Aller 1994; Kristensen and Holmer 2001; Reimers et al. 2013; Aller 2014). The numerous burrows being intensively irrigated by *A. filiformis* induce highly dynamic redox conditions in the upper sediment layers, and this may indeed have facilitated further degradation of terrestrial material than would otherwise be expected in these high-deposition environments. Despite an overall RQ that is close to unity, the high spatial and temporal variation in benthic O_2_ availability implies an extensive microscale variation in the redox condition creating a dynamic mosaic of concurrently ongoing reductive and oxidative microbial and chemical pathways within the surface sediment. This has important implication for the regeneration and the exchange of nutrients.

Given the relatively high mineralization rates and the dynamic redox condition, most N_2_ production in these sediments can presumably be ascribed to denitrification rather than anammox (Rysggard et al. 2004; Trimmer et al. 2013; Devol 2015). The denitrification rate (2.0 *±* 1.1 mmol N m^−2^ day^−1^), as inferred from the net release of N_2_, is in the high end of available measurements for coastal sediments of similar reactivity (Herbert 1999; Eyre and Ferguson 2009; Devol 2015) and could not be sustained by the relatively moderate NO_3_^−^ uptake from the bottom water (0.43 *±* 0.42 mmol N m^−2^ day^−1^). It follows that at least 80 % of the denitrification was coupled to nitrification. Assuming, for the sake of argument, that the organic material being mineralized had an element composition close to Redfield ratio (C–N–P 106:16:1) the DIC release rate would correspond to an ammonification of 2.3 *±* 0.5 mmol N m^−2^ day^−1^. The benthic efflux of NH_4_^+^ was only 20 % of this value (Table 1), implying that a substantial fraction was being nitrified in the sediment. Measured NO_3_^−^ microprofiles indeed documented nitrification just below the sediment interface (Fig. 3). But the activity was patchy, and based on simple Fickian gradient calculations, the nitrification activity at the surface on average amounted to only 0.41 *±* 0.30 mmol N m^−2^ day^−1^ of which >50 % diffused into the bottom water rather than being denitrified. Denitrification therefore must have been sustained to a large extent by nitrification activity in the ventilated *A. filiformis* burrows. The irrigation of oxygenated water through the complex burrows presumably facilitated an efficient oxidation of NH_4_^+^ and supplied NO_3_^−^ for denitrification in the surrounding sediment. Given the burrow geometry and the ongoing irrigation, NO_3_^−^ that in the first case would have been released to the central cavity or the burrows could subsequently be denitrified further downstream, as the water gradually became depleted in O_2_ and passed the burrow system. The activity of *A. filiformis* therefore enhanced the nitrification and the coupled denitrification making the sediment an efficient sink for bioavailable nitrogen. A preferential stimulation of coupled denitrification has previously been documented for ventilated U-shaped burrows of the amphipod *Corophium volutator* (Pelegri and Blackburn 1994, 1996; Rysgaard et al. 1995) and the thalassinidean shrimp *Typaea australiensis* (Webb and Eyre 2004).

The chamber incubations revealed a consistent efflux of PO_4_^3−^ (0.29 *±* 0.26 mmol P m^−2^ day^−1^) that on average exceeded the expected release associated with the degradation of organic material having a Redfield C–P ratio (0.14 mmol P m^−2^ day^−1^). The difference was, however, not statistically significant, and the deposition and mineralization of organic P are not temporally coupled to the benthic release of PO_4_^3−^ (Jensen et al. 1995). It is well established that Fe oxyhydroxides efficiently adsorb PO_4_^3−^, that upon reduction is released, typically in a ratio of 1:3 (Fe–P) (Gunners and Blomqvist 1997). The sediment of Loch Etive has a high content of iron undergoing a dynamic redox cycle (Overnell et al. 1996). It is highly plausible that fauna-induced particle mixing and advection lead to transient reduction of iron oxides in certain areas of the burrows, and a subsequent release of PO_4_^3−^, that was being advected out of the burrow system with the O_2_-depleted water. However, the adsorption and desorption of PO_4_^3−^ at the highly dynamic redox conditions of the burrow environment are complicated, and the net result will be highly dependent on bottom water O_2_ availability and deposition dynamics. Furthermore, riverine sources of non-organic and particle-bound PO_4_^3−^ could contribute to sedimentary inventory of P in Loch Etive (Benitez-Nelson 2000).

The average ratio between the efflux of DSi and DIC is 0.21. This is somewhat higher than the value expected for the degradation of freshly deposited pelagic diatoms, which would be on the order of 0.13 (Brzezinski 1985). The elevated release of DSi could reflect that the material undergoing mineralization is enriched in Si compared to fresh pelagic diatoms (Jahnke and Jahnke 2000) or additional sedimentary supply of Si from various terrestrial sources (Conley 2002) entering complex redox-dependent dissolution kinetics (Aller 2014). In any case, in situ chamber incubations typically provide markedly higher DSi fluxes as compared to exchange rates derived from porewater calculations and shipboard incubations and this has generally been ascribed to bioirrigation (Berelson et al. 2003; Hammond et al. 2004; Tréguer and De La Rocha 2013). The high infauna activity may very well have facilitated a high efflux of DSi in the sediments of Airds Bay.

### Outlook and Perspectives

By combining laboratory-based planar optode investigations with three different in situ approaches for assessing benthic solute exchange, we have provided a unique integrative assessment of benthic carbon mineralization and nutrient regeneration in the coastal, fauna-rich sediment of Loch Etive. The in situ investigations only target a confined period, but demonstrate the extensive temporal and spatial variation in benthic mineralization and derived solute exchange at the seabed. Laboratory-based investigations and discrete sampling have provided important insight into the mechanics of early diagenesis, and such approaches will remain essential in future studies. However, we also need to better appreciate the implications of naturally occurring environmental events, dynamics and spatial variations which too often are ignored by standard measuring procedures. Coastal sediments are highly dynamic and exhibit an extensive variation on several spatial scales, and we still have poor understanding on how this interrelates to the overall biogeochemical functioning of the system. This applies to not only cohesive muddy sediments, which traditionally have attracted most attention, but also permeable sands, fluid beds and hard bottom substrates. Only by fully appreciating this complexity of temporal dynamics and spatial heterogeneity, and as important, by assessing their scales correctly, can we arrive at representative mean values for specific benthic environments. To tackle these challenges, we need to further reinforce our efforts in developing observational platforms and measuring approaches for realising integrated process-oriented in situ studies.

## Electronic supplementary material

Below is the link to the electronic supplementary material.

Supplementary material 1 (AVI 8683 kb)

Supplementary material 2 (AVI 9642 kb)

## Footnotes

1 Including values from areas not affected by fish farming and excluding a previously published laboratory-determined value from Airds Bay of 52.3 mmol m^*−2*^ *day* ^*−1*^ *(Overnell et al.* 1996 *) which we consider to be untrustworthy.*
